# Weight Status and Attentional Biases Toward Foods: Impact of Implicit Olfactory Priming

**DOI:** 10.3389/fpsyg.2019.01789

**Published:** 2019-08-09

**Authors:** Marine Mas, Marie-Claude Brindisi, Claire Chabanet, Sophie Nicklaus, Stéphanie Chambaron

**Affiliations:** ^1^Centre des Sciences du Goût et de l’Alimentation, AgroSup Dijon, CNRS, INRA, Université Bourgogne Franche-Comté, Dijon, France; ^2^Department of Diabetes and Clinical Nutrition, Centre Hospitalier Universitaire de Dijon, Dijon, France

**Keywords:** obesity, attentional biases, priming, odors, cognition, overweight, attention

## Abstract

**Objective:** Numerous studies highlight the involvement of cognitive factors in the development and maintenance of obesity. We aimed to measure attentional biases (AB) toward foods (i.e., the individual tendency to automatically orient one’s attention toward food stimuli) in normal-weight (NW) individuals and those with overweight (OW) and obesity (OB). We evaluated whether implicit or explicit exposure to olfactory food cues could modify AB.

**Methods:** Eighty-five participants with different weight statuses took part in this experiment. We measured AB toward food pictures with an adapted visual probe task and the variations in AB, while participants were primed with olfactory food cues (within-subject design: no odor/low-energy dense food odor/high-energy dense food odor). Odors were non-attentively perceived during session 1 (implicit condition) and attentively perceived during session 2 (explicit condition).

**Results:** Our results highlighted AB toward food pictures, especially when foods were energy dense, regardless of weight status (*p* < 0.001). The olfactory priming effect was only significant in the implicit condition. Participants with obesity had a stronger AB toward foods when they were primed with a non-attentively perceived high-energy dense food odor than with a non-attentively perceived low-energy dense food odor (*p* = 0.02). The trend was reversed for normal-weight participants, while no significant effect was found for participants with overweight.

**Conclusion:** Our results support the hypothesis that an obesity-specific cognitive vulnerability may influence the processing of food-related stimuli and only while food cues are non-attentively perceived. Future research should seek to understand the mechanisms of this phenomenon.

## Introduction

Currently, one of the major goals of public health policy is to induce modifications in individual behaviors in order to improve global health. The main objective is to raise public awareness of unhealthy behaviors, ideally leading the population to adopt a healthier lifestyle. There is a particular focus on diet and, more precisely, food choices. In recent decades, several public policy measures have sought to influence the public with concrete actions, for instance providing consumers with nutritional information or implementing incentive interventions (Programme National Nutrition Santé, 2004; Hill et al., 2008; National Clinical Guideline Centre (UK), 2014; Knai et al., 2018). Subsequent analyses have revealed that these measures often resulted in null or moderate short-term health improvements (Mozaffarian et al., 2018). Research has even demonstrated a boomerang effect, which indicates a need for innovative approaches to diet and behavior modification (Rabia et al., 2006). Some authors link the meager effect of these interventions to the fact that present solutions explicitly and directly target behavior, while food decisions often happen below the threshold of consciousness (Jacquier et al., 2012). However, the conscious and non-conscious mechanisms that motivate food choices are poorly understood. The lack of conclusive data on this subject illustrates the importance of disentangling the mechanisms that underlie food choices in order to improve the efficacy of public health strategies.

The World Health Organization (WHO) defines obesity as a disease in which excess body mass has accumulated to such an extent that health may be adversely affected (WHO | Obesity and Overweight, 2017; WHO/Europe Approaches to Obesity, 2017). The prevalence of obesity worldwide has tripled since 1975; it has been defined as the fifth cause of mortality worldwide. In France, nearly 50% of the adult population is currently considered obese or overweight (Anses - Agence nationale de sécurité sanitaire de l’alimentation, de l’environnement et du travail, 2015). The current perspective on obesity is that it has a multifactorial etiology that involves biological, social, and behavioral aspects. Abundant research has been conducted in order to disentangle the complex mechanisms contributing to the development and maintenance of excess weight. Nevertheless, obesity has thwarted researchers’ efforts to characterize it. In addition, the term “obesogenic environment” is increasingly common in the scientific landscape, implying that environmental factors have also played a part in the current prevalence of obesity (Glanz et al., 2005; Swinburn et al., 2011; Paquet et al., 2017; Townshend and Lake, 2017).

To be able to influence eating behavior, environmental stimuli must (1) be perceived by the sensory organs, (2) undergo cognitive processing, and (3) be considered relevant for individual goals. Each of these three conditions can be processed with separate and variable degrees of attention, from the premises of perception to the feedback concerning related behavior. Indeed, the interaction between an individual and his or her environment is determined by the cognitive processing of environmental information. Accordingly, an investigation into the cognitive processing of food stimuli could contribute to a better understanding of how the environment influences the individual food choices that play a potential role in overweight and obesity. According to the “foraging theory” of Manohar (Manohar and Husain, 2013), attention can be regarded as a low-cost alternative to moving around and physically interacting with the environment before a decision is made to interact physically with the world.

Individuals are constantly flooded with food-related sensory information: visual or auditory messages about nutrition, streets lined with restaurants, food advertising, and so on (Glanz et al., 2005; Swinburn et al., 2011; Paquet et al., 2017). However, our limited processing resources make it impossible to fully capture the perception and processing of sensory information (Chun et al., 2011). In a 2008 review of the literature, Cohen demonstrated that food choices are mostly made automatically and that the influence of the environment on such choices is often insidious, asserting that “excessive food consumption occurs in ways that defy personal insight or are below individual awareness” (Cohen, 2008). This statement highlights the existence of two types of environmental stimuli: non-attentively-perceived food stimuli (implicit exposure) and attentively perceived food stimuli (explicit exposure).

Previous studies comparing those two modes of exposure show that implicitly perceived stimuli have more influence on behavior than attentively perceived stimuli, as they seem to have a different influence on cognitive processes (Hess et al., 2004; Holland et al., 2005; Smeets and Dijksterhuis, 2014). Regarding public health policies, initial studies on nudging (i.e., modifying the environment in a non-invasive manner in order to subconsciously influence behavior) have shown interesting results on food choice. In 2017, a study found that putting participants in a green and leafy environment led to a decrease in the consumption of high-energy food (chili con carne) and an increase in the consumption of low-energy food (salad) (Friis et al., 2017). Nudging approaches are based on guiding consumer toward a different choice without modifying incentives or decreasing the number and probability of options (Thaler and Sunstein, 2009). In this context, we can view priming as a form of nudging, considering that it is defined as the exposure to a stimulus which activates a concept that is then given increased weight in subsequent judgments.

Studies have shown that implicit priming with food odors may influence food choices in adults, the elderly, and in children. Gaillet et al. (2013) and Chambaron et al. (2015) showed the effect of implicitly perceived food odors (pear) on subsequent food choices in normal-weight adults, supporting the hypothesis of food-cue-specific priming effects. In those studies, people were more likely to choose a healthy dessert when they were implicitly primed with a pear odor than in the control condition (Gaillet et al., 2013; Chambaron et al., 2015). In an attempt to stimulate food intake in people with Alzheimer’s disease (AD), Sulmont-Rossé et al. (2018) diffused a meaty odor into the dining room of Alzheimers’ units before lunch. The authors hypothesized that a food odor would trigger food-related mental representations, which in turn may stimulate appetite, willingness to eat, and food intake through implicit processes (priming effect). The results revealed a significant effect, with a 25% increase in meat and vegetable consumption when a meat odor was primed, compared with control condition. Behavioral assessments also showed that residents were significantly more interested in the meal when it had been primed. In addition, Marty et al. (2017) showed that implicitly perceived food odors (low-energy dense food odor – pear – vs. high-energy dense food odor–pound cake) influence food choices intentions in children with normal-weight status and overweight. Children with overweight and obesity were more likely to choose healthy foods than children with a normal weight status when both were implicitly primed with the low-energy dense food odor. The authors hypothesized that the differences in influence of primes were linked to differentiated activation of mental representations depending on weight status. Those results indicate that olfactory priming affects the processing of food choices. Olfactory stimuli are able to draw attention unless they are particularly pleasant or strong (Smeets and Dijksterhuis, 2014), making them appropriate primes to be (1) used in a context of implicit priming and (2) combined with an assessment of cognitive processes. These findings indicate that olfactory stimuli are appropriate for the study of implicit priming effects on individuals’ food choice processing because they seem to influence food choices and the activation of mental representations that underlie those choices. To our knowledge, the effects of implicit and explicit olfactory priming effects have not yet been investigated in studies on eating behaviors.

In order to assess the cognitive treatment of food stimuli, we focused on the attentional selection of visual stimuli involved in the earliest stages of cognitive information processing (Posner, 2016). Attentional selection begins with the initial orienting of attention: the orienting network is focused on the ability to prioritize sensory inputs by selecting a location in space (Posner, 1980). Attentional biases (AB) are cognitive biases, which can be described as automatic adaptive processes enabling the most representative perception of reality with the lowest cognitive cost. They are defined by the fact that certain types of stimuli are more salient for the individual and are consequently more prone to visually attract attention and undergo cognitive treatment. Munneke et al. (2016) characterized salient stimuli in general as stimuli that (1) relate to an individual’s motivation and (2) that are associated with reward. Food stimuli fulfill those two conditions because eating is necessary for survival and is consequently tied to strong individual motivation (Hopkins et al., 2000). In addition, the sensations of pleasure and satiation associated with food consumption activate the dopaminergic system, similar to addictive substances, and are thereby strongly associated with reward (Kringelbach and Berridge, 2009; Alonso-Alonso et al., 2015; Joyner et al., 2017).

Several authors have already investigated attentional biases toward food stimuli, in various populations (normal weight and obese adults, restrained eaters, children, and adolescents) and confirmed that these biases are significant (Mogg et al., 1998; Castellanos et al., 2009; Werthmann et al., 2011; Yokum et al., 2011; Shank et al., 2015; Kemps et al., 2016). These studies show that the intensity of ABs toward food stimuli differs according to, first, situational characteristics such as prandial state: hungrier people display stronger ABs (Mogg et al., 1998; Castellanos et al., 2009; Forestell et al., 2012). Secondly, intensity is modulated by individual characteristics such as weight status (people with overweight or obesity tend to have higher AB, especially when hungry or craving certain foods) (Castellanos et al., 2009; Werthmann et al., 2011; Yokum et al., 2011; Hendrikse et al., 2015), personality traits (attentional impulsivity is correlated with greater ABs toward foods) (Hou et al., 2011), and eating styles (external eaters showing greater AB for example) (Hou et al., 2011). Thirdly, the characteristics of the stimulus can intensify AB, for example, foods that represent a significant source of energy or of danger (Garcia-Burgos et al., 2017).

Moreover, in 2016, Kemps et al. observed that, in adults, the intensity of ABs toward food stimuli could be used to predict weight gain 1 year after the first measurement. Werthmann et al. also reported the predictive effect of AB on weight loss but in a study of weight loss in obese children (Werthmann et al., 2015). Finally, Cohen (2008) indicated that increased salience could enhance the probability that a food would be chosen. In sum, attentional biases appear to be a key point in decision-making, and food choices are not an exception to the rule.

The above studies support the hypothesis that there are food-specific ABs that may differ in relation to weight status and that their presence can be linked to weight evolution. In an obesogenic environment, such biases could denote a cognitive vulnerability, making adults with obesity more prone to the cognitive treatment of food stimuli and thus more likely to seek, choose, and overconsume foods, especially when they are energy dense. Combined with the previously described obesogenic environment, this cognitive bias is an unsettling factor for the energy intake/expenditure balance, making unhealthy foods more salient, and pushing individuals to make unhealthy food choices (Cohen, 2008).

The aim of this study was to explore attentional biases toward food stimuli in normal-weight individuals, and in those with overweight or obesity. In order to investigate the influence of environmental food cues on such biases, we assessed attentional biases while implicitly and explicitly priming with olfactory food cues, i.e., odors signaling a high-energy dense (HED: pound cake odor) or a low-energy dense (LED: pear odor) food.

The first objective of our experiment was to characterize attentional biases in people with different weight statuses. We aimed to replicate previous results with the hypothesis that people with obesity would present stronger ABs toward foods, especially when these foods were energy dense, than participants with normal weight. It was assumed that participants with overweight would present an intermediate model. Secondly, we intended to investigate the specific effect of food cues (odors suggesting HED or LED foods) on ABs for each weight status group. Our hypothesis was that the olfactory prime type would have food-cue priming effects that would differ between groups. The third objective was to evaluate the impact of implicit vs. explicit exposure to food odors on the visual processing of food cues in order to establish whether implicitly perceived odors would have a stronger influence on ABs.

## Materials and Methods

### Participants

Overall, 107 adults aged 25–59 years old were included in this study. We divided our sample into three categories of weight status based on body mass index (BMI, kg/m^2^, Nuttall, 2015; Komaroff, 2016): normal-weight (NW; 18.5 ≤ BMI < 25, *n* = 41), overweight (OW; 25 ≤ BMI < 30, *n* = 35), and obesity (OB; BMI ≥ 30, *n* = 31).

Participants were recruited from the population registered in the Chemosens Platform’s PanelSens database. This database complies with national data protection rules and has been vetted by the appropriate authorities (Commission Nationale Informatique et Libertés – CNIL – 135 *n* = 1,148,039).

Exclusion criteria were chronic disease (such as diabetes, hypertension, or any type of cardiovascular disease), specific diet (such as vegetarian, gluten free, fat free, salt free), medical treatment that may affect mental awareness, bariatric surgery history, pregnancy, anosmia, or chronic sinusitis. Furthermore, we instructed participants to postpone the date of their session if they were feeling symptoms of the flu or a cold to avoid the possibility of decreased olfactory capacities. Moreover, we checked participants’ olfactory capacities with the European Test of Olfactory Capacities (ETOC – Thomas-Danguin et al., 2003) in order to ensure that the sample had a proper sense of smell (detection and identification) while priming them with low-concentrated odors.

The study was conducted in accordance with the Declaration of Helsinki and was approved by the local ethical committee (Comité d’Evaluation de l’Ethique de l’Inserm – CEEI, File number IRB 0000388817-417–Project number X 467). This research study adhered to all applicable institutional and governmental regulations concerning the ethical use of human volunteers.

Written informed consent was obtained from participants before their participation, though they came to the sessions under a false pretense (i.e., to participate to a computerized experiment on picture categorization). At the end of the experiment, participants were entirely debriefed and told the real purpose of the study. In return for their participation, the participants received a €20 voucher at the end of the two sessions.

### Session

Our study was held in two sessions, about 10 days apart. Participants were told to come to the laboratory, under a false pretense, at lunchtime (12:00). They were instructed to refrain from eating, smoking, and drinking anything, except water, for 3 h before the session. They were also asked not to wear any perfume/scented cosmetics on the day of the sessions.

During the two sessions, participants completed The Food Adapted-Visual Probe Task (FA-VPT), while being primed with implicit (session 1) and explicit (session 2) food odors (Marty et al., 2017). At the end of the second session, participants answered a psychological assessment questionnaire.

Each session began and ended with a short questionnaire about the level of hunger [*“On a scale of 1 (not hungry at all) to 10 (very hungry), how hungry do you feel right now?”*] mode of transportation to come to and leave the laboratory (decoy questions to distract participants from the real purpose of the study). Before computing, the FA-VPT dominant hand, age, and sex of the participants were measured. Participants were seated in front of a computer screen and instructed not to move their head during the task. During the experiment, the instructions related to the task were given through a headset-microphone in order to justify the presence of the headsets used for olfactory priming.

### Food Adapted-Visual Probe Task

In order to assess attentional biases toward food, we adapted the Visual Probe Task (MacLeod et al., 1986), which is classically used for measuring attentional biases (Mogg et al., 2003; Petrova et al., 2013; Iacoviello et al., 2014; Price et al., 2016).

**Figure 1:**
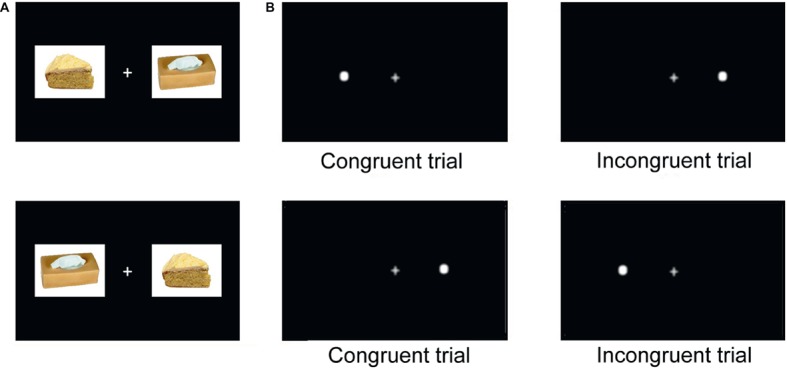
Food adapted-visual probe task trials. **(A)** Stimuli pairs appeared during each block in the two orders (two presentations). **(B)** The white dot appeared either on the left or on the right side of the fixation cross (two presentations). This gives four presentations per stimuli pair within each block 2 (picture position) × 2 (probe position). When the dot replaces the critical stimulus, the trial is considered congruent. When it replaces the control stimulus, the trial is considered incongruent.

A fixation cross appeared on the center of the screen at the beginning of each session and remained on screen throughout. After 500 ms, two stimuli appeared simultaneously on the right and left side of the fixation cross. Immediately after, a probe (white dot, Arial, 50) replaced one of the two images and remained on screen until the participant indicated its location (left or right, see Figure 1). No feedback was given to the participant in order to avoid interference in attentional processes (Kluger and DeNisi, 1996). Then, the following trial began 500 ms after a manual response was given.

In our experiment, the task began with a brief training session comprising 20 trials using five pairs of neutral stimuli (pictures of everyday life objects from FoodPics database, Blechert et al., 2014) appearing four times each. This training aimed to familiarize participants with the repetition of image pairs and to ensure task comprehension. After the initial training session, participants completed three blocks, punctuated by short breaks (3 min). Blocks were comprised of 168 trials each: 42 pictures pairs appeared four times in a random order (see Figure 1). Participants were instructed to indicate the location of the dot by pressing a key on an AZERTY keyboard (A for left and P for right). The dot appeared during each trial. It replaced the critical stimulus in 50% of trials (congruent trial), and the control stimulus in 50% of trials (incongruent trial). Reaction times (RT) for each trial were recorded in milliseconds.

### Stimuli

In order to assess attentional biases toward food stimuli, we chose to use visual stimuli. They were pictures selected from the database FoodPics (Blechert et al., 2014). A first sorting was made in order to exclude complex pictures and culturally inappropriate foods for a French sample. Pictures were matched so as to obtain three types of pairs of stimuli:

1. HED-CTL (high energy dense-control): Image of a high-energy dense food paired with a neutral control image (everyday life objects from FoodPics database) in order to study attentional biases toward high-energy dense foods.
2. LED-CTL (low energy dense-control): Image of a low-energy dense food paired with a neutral control image (everyday life objects from FoodPics database) in order to study attentional biases toward low-energy density foods.
3. HED-LED (high energy dense-low energy dense): Image of a high-energy dense food (muffin, chocolate bar) paired with an image of a low-energy density food (apple, watermelon slice) in order to observe how attention is oriented when presented with two food stimuli with contrasting energy densities.

The perceptive properties of each pair of pictures were matched – color, size, brightness, within-object contrast, spatial frequency, and complexity. The perceptive dataset was standardized and used to calculate the Euclidian distance matrix between pairs of pictures. Then, pairs of pictures with similar features were chosen and subsequently validated by three experimenters.

**Figure 2:**
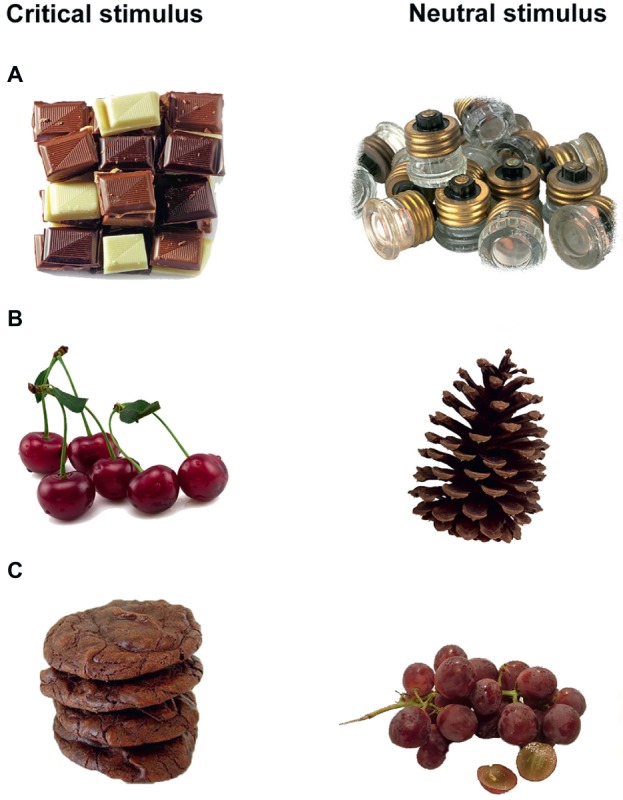
Picture pairings. **(A)** Example of HED-CTL, designed to assess AB toward high-energy dense foods. **(B)** Example of LED-CTL, designed to assess AB toward low-energy dense foods. **(C)** Example of HED-LED, designed to assess where attention is oriented between two stimuli of contrasted energy density.

Fourteen pairs were formed for each pair type. Each pair contained one critical stimulus and one matching control stimulus. For odor-congruency reasons, we chose to use only pictures of sweet foods, with a contrast in energy density to reflect the continuum of healthy-unhealthy foods. An online survey was created a posteriori in order to ensure the matching of food pictures among HED-LED pairs. We assessed perceived hedonic value (*n* = 125), appetence (*n* = 86), perceived healthiness (*n* = 69), perceived energy density (*n* = 69), categorical representability (*n* = 48), and environmental occurrence (*n* = 46), which were evaluated for each pre-selected picture representing food by an independent participant sample. As the picture database was not created for a French sample, we also checked that the foods in the chosen pictures were easy to recognize for French participants. Furthermore, food picture pairings (HED-LED) were chosen according to perceived differences in healthiness (*n* = 69) and in energy density (*n* = 69) from the online survey. See examples of resulting picture pairs in Figure 2.

### Olfactory Priming Paradigm

During the task, participants were primed with different olfactory food stimuli, according to the paradigm developed by Marty et al. (2017). Throughout the sessions, oral instructions for the FA-VPT were transmitted to participants through a headset. Participants wore three successive headsets whose microphone had been odorized beforehand. Experimenters switched the headsets during break times, unbeknownst to participants.

**Figure 3:**
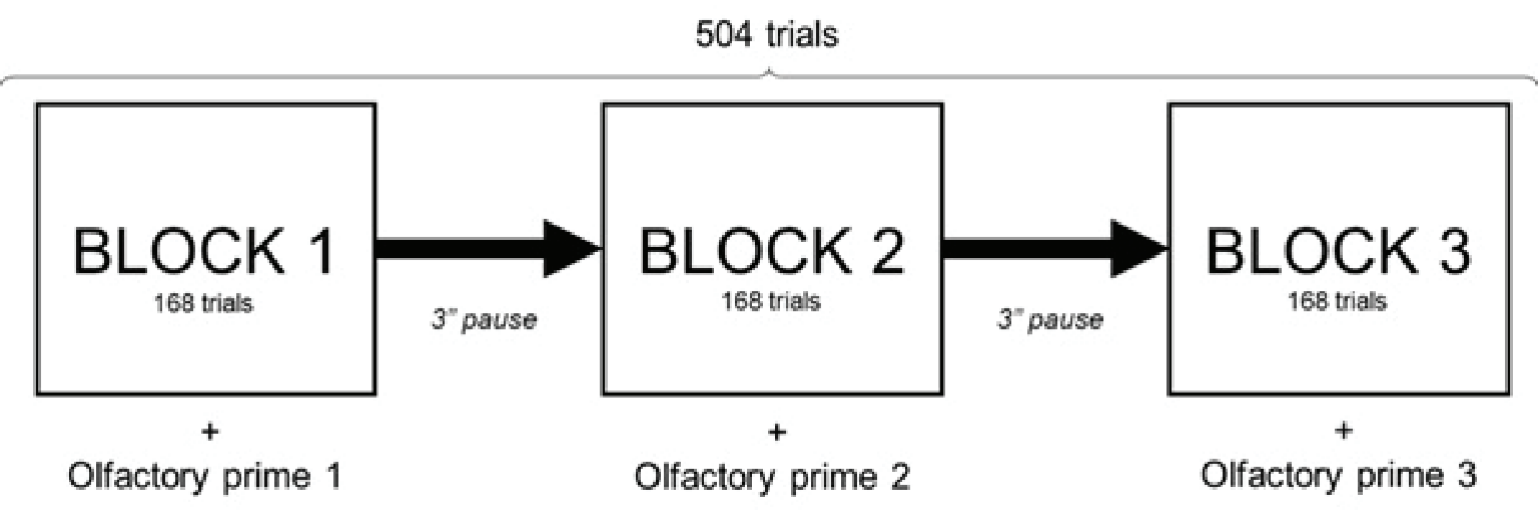
Design of the olfactory priming paradigm within Food adapted-visual probe task. Visual Probe Task setting for each session, olfactory prime type order determined by William Latin square design to balance the order and first order carryover effects of the olfactory prime types.

This experiment comprised three different olfactory prime type conditions: fruity odor (pear odor, priming low-energy dense food), fatty-sweet odor (pound cake odor, priming high-energy dense food), and control condition (no odor). As each session comprised three FA-VPT “blocks,” experimenters changed the headsets during the 3-min pauses out of participants’ sight, in order to prime them successively with the three olfactory prime types (pound cake, pear, none). A William Latin Square design was used to determine the order of the olfactory prime presentation in order to balance the order and first-order carryover effects of the olfactory prime types (Figure 3). For each olfactory prime, participants performed 168 trials of the FA-VPT consisting in 14 pairs of images per type presented according to the four modalities described in Figure 1.

During session 1, participants were non-attentively exposed to the olfactory primes (implicit session) while performing the FA-VPT: they were not told about the odors on the microphones of the headset, and an investigation questionnaire filled out at the end of session confirmed that the odors and headset changes were not perceived. During session 2, participants received information about the presence of “some odorized foam” on the microphone of their headset before they performed the FA-VPT (explicit session).

Afterwards, participants responded to the Questionnaire for Eating Disorder Diagnosis (Mintz et al., 1997, French translation from Callahan et al., 2012), for the detection and exclusion of participants suffering from eating disorder according to the Diagnostic and Statistical Manual of Mental Disorder, fourth revised edition (DSM IV TR, American Psychiatric Association, 2000).

At the end of the two sessions, participants were debriefed. They were told the real purpose of the study and given details about the experimental design (hypotheses, implicit priming during session 1, and odor types).

To summarize, four variables of interest were studied in this experiment: weight status (normal-weight, overweight, and obesity), pair type (HED-CTL, HED-LED, and LED-CTL), mode of exposure (implicit and explicit), olfactory prime type (fatty-sweet odor: pound cake, fruit odor: pear, none as control). This design yielded nine experimental conditions (pair type × olfactory prime type, 3 × 3) for each mode of exposure (implicit and explicit). Participants were exposed to each possible condition.

### Statistical Analysis

First, we used medians to summarize reaction times measured in each condition, for congruent trials on the one hand, and for incongruent trials on the other hand. Medians were calculated over 28 values (14 pairs × 2 presentations). We then computed RT bias scores by subtracting the reaction time in congruent trials from the reaction time in incongruent trials, in each individual for each condition, as suggested by Price et al. (2015): RT bias score = RT for incongruent trials − RT for congruent trials. The orienting of attentional bias expressed as the bias score was used as the dependent variable in our study.

A positive RT bias score (bias score > 0) indicated an attentional bias toward the critical stimulus. A negative RT bias score (bias score < 0) indicated an avoidance attentional bias for the critical stimulus (MacLeod et al., 1986). One normal-weight participant was excluded at this step because of extreme avoidance (5 values over 9 less than −100 ms in the implicit condition, see Supplementary Material data for details).

Statistical analysis was performed with R.3.4.3 software (R Development Core Team) using linear mixed models (nlme package v. 3.1-131) (Pinheiro and Bates, 2017) to explain the attentional biases expressed in bias scores. Specific contrasts were subsequently tested using the contrast package. The significance threshold was set at 0.05.

Previous work on implicit olfactory priming (Gaillet et al., 2013, 2014; Chambaron et al., 2015; Marty et al., 2017) was not designed to compare implicit and explicit conditions but focused on the assessment of implicit olfactory priming effects. In the present study, the same type of analyses were computed for the implicit on the one hand and explicit conditions on the other hand to stay in line with previous work: the initial model for each mode of exposure involved all three fixed factors (weight status × pair type × olfactory prime type) and all interactions up to order three, with the random individual factor. Then, the non-significant terms were removed unless they were involved in a significant higher order term.

In order to check for differences in participants’ characteristics between weight status group, we used ANOVAs for quantitative variables (age, BMI, level of hunger, hunger level before session, and hunger progression) and chi-square tests for qualitative variables (sex and level of education). When a significant difference was detected, between-group comparisons were performed with *t*-tests and chi-square tests, respectively, for quantitative and discrete variables.

## Results

### Participant Characteristics and Exclusion

**Figure 4:**
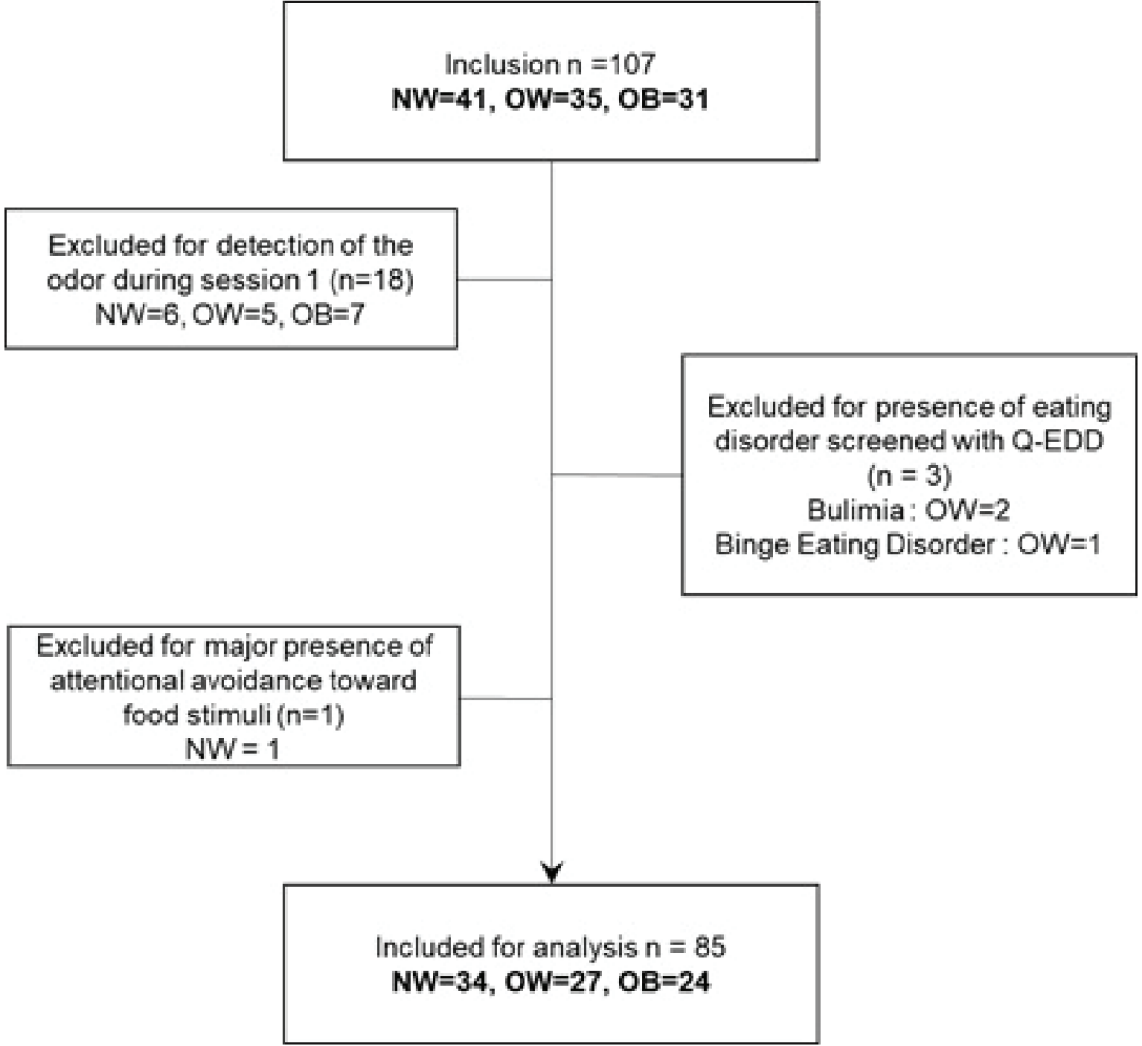
Flowchart of exclusions. NW, participants with normal weight; OW, participants with overweight; OB, participants with obesity.

Participants who indicated that they had noticed a food odor in the questionnaire at the end of session 1 were excluded from the study in order to ensure that every participant was non-attentively exposed to the olfactory cues. Three additional participants were excluded because their answers to Q-EDD indicated that they were suffering from an eating disorder according to the DSM-IV TR (Mintz et al., 1997; Callahan et al., 2012). The European Test of Olfactory Capacities found no specific impairment in the sample. The flowchart for exclusion criteria can be found in Figure 4.

After the exclusion criteria were applied, 85 participants (34 participants with normal weight, 27 with overweight and 24 with obesity) remained for analysis. See sample characteristics in Table 1.

**Table 1:** Participant characteristics.

	Weight status		
	Normal-weight (NW) *n* = 35 (41%)	Overweight (OW) *n* = 26 (31%)	Obesity (O) *n* = 24 (28%)
Age (y): *p* = 0.03	35^a^ (*6.8*)	39^b^ (*7.9*)	40^b^ (*10.3*)
BMI (kg/m^2^): *p* < 0.001	22^a^ (*1.6*)	27^b^ (*1.2*)	34^c^ (*4.2*)
Hunger level before session (1–10): *p* = 0.04	6.1^b^ *(2.0)*	5.4^b^ *(2.1)*	4.5^a^ *(2.6)*
Hunger progression: *p* = 0.24	0.4 *(1.3)*	0.2 *(1.5)*	1.03 *(2.2)*
Sex: *p* < 0.001			
Women	17^b^ (49%)	14^b^ (54%)	21^a^ (88%)
Men	18^b^ (51%)	12^b^ (46%)	3^a^ (17%)
Level of education: *p* = 0.15			
< Bachelor’s degree	7 (20%)	8 (31%)	10 (42%)
Bachelor’s degree	14 (40%)	12 (46%)	11 (46%)
> Bachelor’s degree	14 (40%)	6 (23%)	3 (12%)

*Quantitative variables expressed as mean (SD)*.

*^a, b, c^Superscript letters are associated to values (means or numbers), same letters indicating that the difference between values is not significant p > 0.05*.

The sample was divided into three groups according to weight status; BMI ranged from 18.75 to 45.67 kg/m^2^.

Participants were 25–59 years old. Participants with NW were significantly younger than participants with OW (−4.5 years, *p* = 0.04) and participants with OB (−5.0 years, *p* = 0.02), but the two latter groups’ age did not differ (*p* = 0.78). These variations are consistent with the observation that weight excess increases with age INCA 3 (2017) Evolution des habitudes et modes de consommation, de nouveaux enjeux en matière de sécurité sanitaire et de nutrition | Anses - Agence nationale de sécurité sanitaire de l’alimentation, de l’environnement et du travail, sd.

At the beginning and at the end of the sessions, a Likert scale was used to evaluate hunger level: “On a scale of 1 (not hungry at all) to 10 (very hungry), how hungry do you feel right now?” Participants with obesity expressed less hunger at the beginning of the sessions than normal-weight participants (*p* = 0.012). No differences were observed otherwise, and the progression of the feeling of hunger (calculated by subtracting hunger before experiment to hunger after experiment) was similar in all three groups.

Our sample comprised more women than men (61%), which is typical of non-clinical trials involving people with overweight and obesity. The sex ratio in the obesity group was significantly different from the overweight (*p* = 0.002) or normal-weight groups (*p* = 0.001). The normal-weight and over-weight groups were not different in terms of sex ratio (*p* = 1). Moreover, no difference between groups was found concerning level of education.

### Implicit Mode of Exposure

In the implicit condition, weight status × olfactory prime type was significant [*F*(4, 651) = 2.73, *p* = 0.02], while other interactions were non-significant. Regarding the main effects, only pair type was significant [*F*(2, 651) = 20.61, *p* < 0.001].

**Figure 5:**
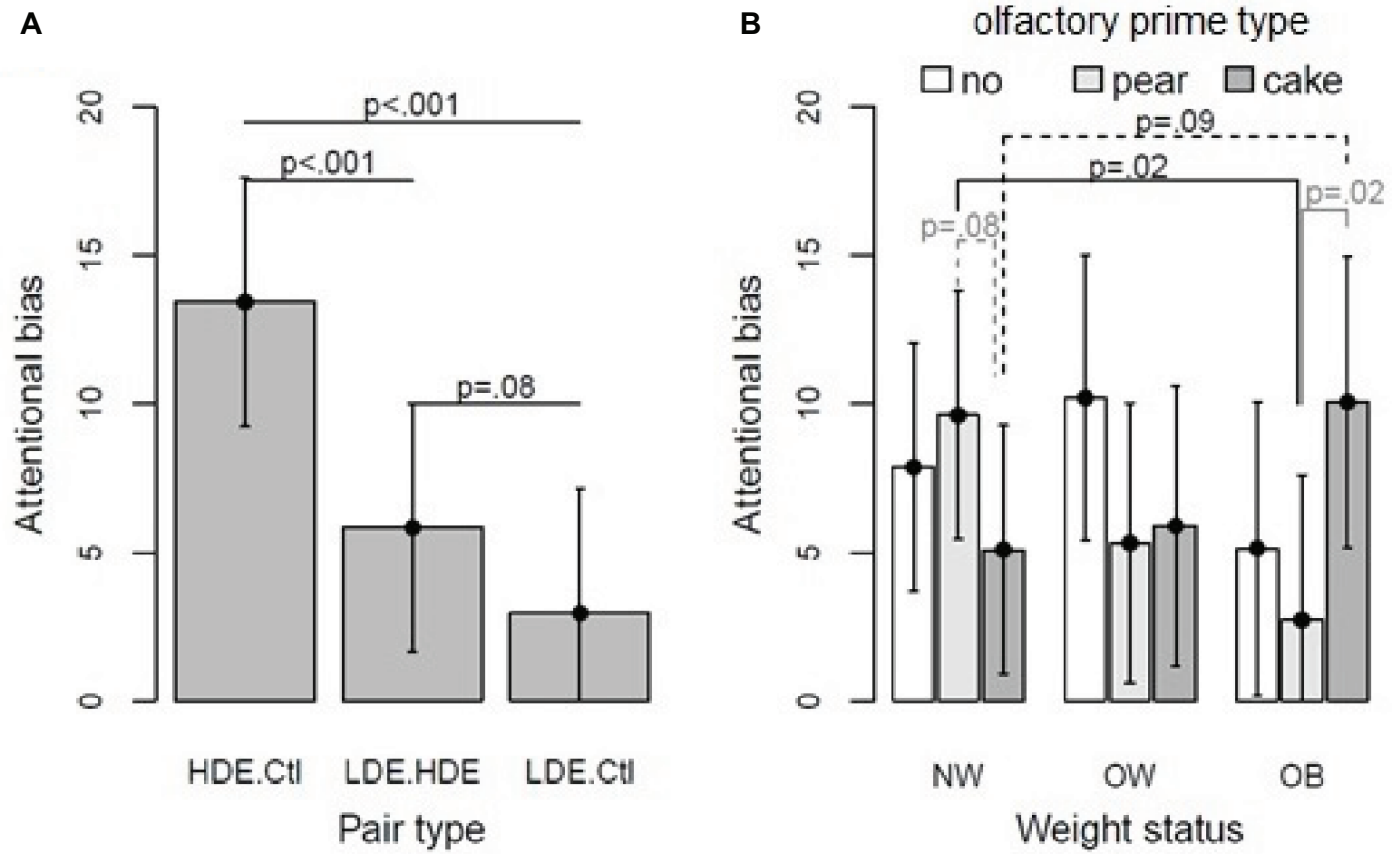
**(A)** Predicted attentional biases and 95% confidence intervals in the implicit condition as a function of pair type (over weight status and olfactory prime type). Linear mixed model, pair type effect *p* < 0.001. **(B)** Predicted attentional biases regarding weight status and olfactory prime type within implicit condition (predictions over the pair type). Linear mixed model, weight status × olfactory prime type, *p* = 0.02.

Then, non-significant terms were removed from the model, and contrasts were assessed to interpret significant effects and interactions. Regarding weight status × olfactory prime type interaction (Figure 5B), bias scores in OB were higher with the pound cake odor than with the pear odor (+7.36 ms, *p* = 0.02) and were higher in NW than in participants with OB exposed to pear odor (+6.91 ms, *p* = 0.02). Moreover, bias scores tended to be higher with pear than with pound cake odors in NW (+4.52 ms, *p* = 0.08), and they also tended to be higher in OB than in NW when exposed to pound cake odor (+4.97 ms, *p* = 0.09).

For the pair type effect, bias scores for HED-CTL were 7.58 ms higher than for HED-LED (*p* < 0.001) and 10.47 ms higher than for LED-CTL (*p* < 0.001); bias scores for HED-LED were 2.89 ms higher than for LED-CTL (*p* = 0.08). For details, see Figure 5A.

### Explicit Mode of Exposure

In the explicit condition, only pair type revealed a significant effect [*F*(2, 648) = 23.0, *p* < 0.001]. Attentional biases regarding pair type presented the same pattern as in the implicit condition analysis: bias scores for HED-CTL were 10.81 ms higher than for LED-CTL (*p* < 0.001) and 8.69 ms higher than for HED-LED (*p* < 0.001), but bias scores for HED-LED were only 2.12 ms higher than bias scores for LED-CTL (*p* = 0.20). In the explicit condition, the interaction of weight status and olfactory prime type was not significant (*p* = 0.69).

A model gathering implicit and explicit conditions showed no significance regarding the interaction between mode of exposure, weight status, and olfactory prime type (*p* = 0.14).

## Discussion

The aim of our study was to explore attentional biases toward food stimuli in normal-weight, overweight, and obese adults. We investigate the influence of olfactory food cues (odors signaling a high-energy dense or a low-energy dense food) on such biases, in an implicit and in an explicit mode of exposure.

### Pair Type Effect

In both implicit and explicit conditions, we found a highly significant pair type effect. Attentional biases toward low-energy dense foods were significantly lower in all participants than toward high-energy foods, regardless of weight status. This finding is consistent with the idea that high-energy foods are more appealing because of their rewarding quality (Kringelbach and Berridge, 2009; Kemps et al., 2016; Joyner et al., 2017). Nevertheless, even if they are comparatively less attractive, foods that are less energy dense also create incentive because they are edible and can be seen as providing positive reinforcement.

To our knowledge, this is the first study to assess the initial orienting of attention between two types of food stimuli in adults. Throughout the experiment, the participants’ attention was oriented toward pictures of foods, especially energy dense foods. In the HED-LED pair, the participants’ attention was oriented toward HED foods. For this pair type, bias score was on average lower than for the HED-CTL pair, which could illustrate an interference effect. Indeed, attention is automatically allocated toward food stimuli. When another food is present, the stimuli may compete for attentional resources more than when food is presented at the same time as a neutral stimulus. This could induce an interference latency in attentional orienting, which would explain the reduced attentional biases in the HED-LED pair-type condition (Posner, 1980).

When two food stimuli are competing to attract attentional resources, the orientation toward HED food is weaker than when it is the only food stimulus in the visual field, possibly reflecting a disturbance in the automatic orientation of attention. In this particular case, we can hypothesize that attentional orienting is more driven by external than endogeneous elements: “where should I look when there is food everywhere?” Indeed, a human being is more prone to look for food in its environment when resources are unpredictably located in space and time (Ohman et al., 2001).

Manohar and Husain (2013) draws a parallel between visual attentional orienting in human beings and animal models of foraging: visual attention is driven by an “engage or search strategy”, thus guided toward uncertain elements (search) or toward rewarding elements (engage). According to this theory, one’s attention engages in the current location or moves to another subjectively more rewarding location (Manohar and Husain, 2013). In the current experiment, participants oriented their attention toward foods, especially energy dense foods, with an intermediate pattern of bias scores for stimuli pairs in which two food pictures appeared. Because high-energy dense foods are rewarding, they are visually salient (Mogg et al., 2003; Cohen, 2008; Alonso-Alonso et al., 2015). Here, Manohar’s theory is applied to the orienting of attention for foods in human adults, as individuals may tend to search for the most rewarding foods (i.e., high-energy dense, palatable foods) (Alonso-Alonso et al., 2015). The automaticity of this process can be explained by the fact that anticipating a stimulus leads to the devaluation of less rewarding stimuli (Flaherty, 1982; Alonso-Alonso et al., 2015).

In an environment where food is abundant, we can speculate that orienting of attention is focused on high-energy dense foods first in order to ensure that these foods are located in space, in order to satisfy a potential physiological need. It can also be supposed that attention is oriented toward the most appropriate choice for the specific motivations of the individual. In sum, we suppose that there is an early prioritization of high-energy dense food stimuli sources, which could explain the lack of clear differences in attentional orienting between participants with normal weight, overweight, and obesity. Our results on this aspect were not conclusive, but they open the way to future research in this field.

### Implicit Olfactory Priming Effects

To our knowledge, this is the first study to assess the effects of olfactory priming on adults with overweight and obesity while comparing implicit and explicit exposure to food odors. Our results indicate that non-attentively perceived food odors influence the cognitive processing of food stimuli, as previously shown by Marty et al. (2017), Chambaron et al. (2015), and Gaillet et al. (2013) but attentively perceived odors had no effect.

In the implicit condition, the attentional biases of participants with normal-weight and obesity differed in intensity depending on the olfactory prime type. Indeed, attentional biases were significantly higher for participants with obesity when they were primed with a non-attentively perceived pound cake odor (signaling HED foods), than when primed with a non-attentively perceived pear odor (signaling LED foods). These odors may have activated different food-related concepts, in turn leading the participant to orient their attention to food more. Furthermore, this activation seemed to be specific to obese weight status. Normal weight participants showed a reversed tendency: when odors were non-attentively perceived, attentional biases for NW were higher during pear odor priming than pound cake odor priming.

While attentional orienting toward foods of all kinds is driven by non-attentively perceived HED food odors for people with obesity, it is not the case for normal-weight participants. This observation is in line with incentive sensitization theory (Joyner et al., 2017), as OB participants seem to be more affected by HED food olfactory cues than NW participants: an environment abundant in high-energy dense foods might modulate the cognitive processing of food cues by making high-energy dense foods more salient, thus more likely to be consumed. In line with the literature in the domain of food priming (Cohen, 2008; Gaillet et al., 2013; Chambaron et al., 2015), the differences in reaction patterns to food odors observed between adults with obesity and normal-weight adults could help to explain the origin of energy dense food choices that contribute to obesity.

When primed with a pound cake odor, adults with obesity seemed to be more orienting their attention toward foods, whereas attentional biases were reduced when they were primed with a pear odor. Marty et al. (2017) used the same implicit olfactory priming paradigm to show that exposure to an implicit pear odor led to more LED food choices in children with obesity than in children with normal weight and found no difference in food choices when the children were primed with a pound cake odor. The differentiated effect of a non-attentively perceived pear odor in obese adults and children, on attentional biases or food choices, respectively, raises several interesting questions: (1) are there different processes activated by the same olfactory prime in children and adults with obesity? And (2) do odors target several processes in different ways, according to the cognitive processing temporality of the olfactory and visual information underlying food choices? Moreover, we can wonder how mental representations of foods are built and subject to environmental cue alteration through childhood to adulthood. Those are future steps for research in olfactory priming.

The difference in AB following implicit HED or LED food odor priming in individuals with different weight status could be related to the activation of different cognitive representations. Doyen et al. (2014) suggested that the features common across procedures include (1) experimenters presenting a prime stimulus, (2) the prime activating an internal representation, (3) the activated representation influencing other representations (this distinguishes semantic or associative priming from repetition priming), and (4) activated representations leading to changes in behavior.

Nevertheless, our findings support the hypothesis that the environment has a differentiated effect on cognition in people with normal-weight and those with obesity but only under certain conditions. Indeed, our results support the idea that attentional biases are influenced by situational characteristics (Garcia-Burgos et al., 2017), which in our case were the olfactory environment.

### Lack of Significance in Explicit Priming Condition

Research suggests that implicitly stimulating participants is more effective for targeting automatic processes than explicit information (Marteau et al., 2012). Accordingly, the effects of priming were only visible in implicit priming conditions in the present study. In explicit priming, the processing of olfactory cues may have been hampered by the participants’ awareness. We hypothesize that participants might have developed cognitive strategies taking the form of a response bias, which lead to different scores in implicit and explicit conditions. This dichotomy, known as the Hawthorne effect, (McCambridge et al., 2014), is typical when information about the experiment is provided directly rather than indirectly. Indeed, participants who were aware of the olfactory priming in session 2 may have in a certain way guessed at the aim of the experiment, which consequently activated mental representations or goals likely to modify their behavior.

### Attentional Bias Regarding Pair Type and Weight Status

The present study is also the first to compare attentional orienting toward food stimuli in people with normal-weight and people with obesity, using three different food-related stimuli pairs. Stimuli were accurately and meticulously paired according to their visual properties (e.g., size and brightness) and consumer-related features (e.g., perceived hedonic value and perceived health value). This pairing process reinforces the relevance of the Food Adapted-Visual Probe Task measurement of attentional biases.

Without priming, there was no significant indication in our results that there is a specific orienting of attention toward foods in people with overweight/obesity compared with normal-weight people. This contradicts previous reports wherein individuals with higher BMIs were more prone to focus their attention on foods (Nijs et al., 2010; Werthmann et al., 2011; Yokum et al., 2011). On the contrary, our results support other studies showing that weight status has no effect on attentional biases toward foods (Ahern et al., 2010) or only under certain conditions (Castellanos et al., 2009; Kemps et al., 2016) such as the implicit olfactory environment effect found in our study. Contradictory findings in the literature can be linked to the use of multiple designs within the use of a Visual Probe Task. Hence, one of the main challenges when developing the FA-VPT was to ensure that improving the relevance of the paradigm would not negatively affect the comparability with other studies using visual probe task paradigms in the food domain or the results of our experiment.

### Overweight Participants

We did find some effects demonstrating differences between normal-weight participants and those with obesity. Concerning participants with overweight, no specific orienting of attention emerged from our data. Future analysis of the psychological assessment questionnaires completed by participants at the end of session 2 might contribute to a clarification of weight-related cognitive profiles. Differences in personality, eating habits, and lifestyle may explain, at least in part, the lack of effect in the overweight group.

The present study tried to replicate the effects of food cues on cognition in a laboratory setting, in order to assess attentional biases in adults of various weight statuses. Our results demonstrated that the presence of non-attentively perceived food stimuli has a differentiated influence on how people with normal-weight and obesity process visual food-related stimuli; however, further research is needed to clarify the development and processing of food-related cognition.

### Limitations

Our experimental setting (two stimuli appearing on a computer screen simultaneously) does not reflect real-life situations. One must be careful while interpreting these findings, as context plays a major role in food perception (Meiselman, 1992; de Castro, 2000; Boutrolle and Delarue, 2009).

We only used sweet stimuli (odors and pictures), as sweet foods are more prone to drive cravings, and have specific properties on dopaminergic system, compared to other food types. (Alonso-Alonso et al., 2015). In addition, sweet foods pictures are more commonly liked and induce a stronger desire to eat (Blechert et al., 2014). Sweet odors were chosen in order to stay in line with the protocol used in Marty et al.’s work. It would have been more difficult to obtain a set of equally liked savory stimuli (odor and pictures) with varying energy densities to study the effect of savory food olfactory primes on attentional biases toward savory food stimuli. Thus, one must then be careful before generalizing those results to other food types.

Furthermore, we only used sweet food stimuli (pictures and odor type), in order to compare the effect of food primes on attentional biases toward foods. Because participants came to the laboratory at lunchtime, when French people typically eat a savory main dish followed by a sweet dessert, such primes might be only partly relevant. Moreover, sweet foods are more likely to trigger cravings and have specific effects on the dopaminergic system (Alonso-Alonso et al., 2015). Therefore, it is difficult to generalize these results to other food types and meal periods.

Due to an experimental constraint inherent to the protocol, the explicit priming condition always took place during session 2. Indeed, planning explicit exposure before implicit exposure can create priming effect between the two sessions and influence the effect of implicit priming. As we needed to assess implicit and explicit priming effects on individuals with potential different sensitivity to these cues, we could not use a between-subject design. A second study with a wider focus on the phenomenon occurring in the explicit condition and the effects of explicit priming of pear and pound cake odor should be conducted. Such work would improve our understanding of how the explicit mode of exposure affects cognition and how those odors are cognitively processed to block food-cue priming effects.

In addition, the FA-VPT could not provide precise measure of whether observed attentional biases were the result of speeded detection of food stimuli or of slowed disengagement of those stimuli. Our results highlighted the attentional saliency of foods influenced by implicitly perceived olfactory cues in individuals with normal-weight, overweight, and obesity. Thus, distinguishing those aspects, for instance by adding trials with neutral pictures pairs (Koster et al., 2006; Cisler et al., 2009), or by using eye-tracking (Werthmann et al., 2011) could provide information on how olfactory cues can influence the cognitive processing of food stimuli.

Finally, reaching an acceptable sample size was challenging because it is difficult to establish specific contact with people with obesity. Even if the INCA3 report states that more than one person out of six is obese in France (Anses - Agence nationale de sécurité sanitaire de l’alimentation, de l’environnement et du travail, 2015), we had difficulty including this population in our research even while recruitment information were intentionally non-specific to avoid the interference of stigma (Major et al., 2014). In addition, we excluded patients with chronic diseases in order to ensure that sensory capacities of participants were not altered by any pharmaceutical treatment. Unfortunately, chronic conditions such as diabetes and cardiovascular diseases are frequent in the population with obesity (Barnes, 2011).

## Conclusion

This study provides new perspectives on the visual processing of food stimuli in individuals with different weight statuses when they are exposed to certain olfactory conditions.

Our first objective was to investigate the differences in attentional orienting for people with normal weight, overweight, and obesity. Our experiment confirms strong attentional biases toward foods (especially HED) and adds evidence that weight status has no significant effect on patterns of attentional orienting to foods. This could imply that this process is similar for all individuals, regardless of weight.

Concerning our second objective, we showed the effects of implicit olfactory food priming and its influence on attentional processing of visual food stimuli for the first time in adults with various weight statuses. Our results indicate that implicit priming influences the orientation of attention toward foods, and they support the hypothesis that individuals with obesity have specific cognitive sensitivity to pleasant olfactory food cues. This difference was only found during implicit exposure to food odors, supporting our third hypothesis that non-attentively perceived food cues affect cognition more than attentively perceived food cues.

In addition, automatic processes such as attentional biases are thought to be promising levers to target behavior (Marteau et al., 2012). Studying such biases could lead to a better understanding of the development of maladaptive food choices, and, as shown by Kemps et al. (2016), attentional biases can be trained, potentially guiding people toward healthier food choices. Targeting the cognitive processing of food stimuli could thus be a promising means to encourage healthier behaviors throughout the population.

Further research could lean toward disentangling the link between the cognitive processing of food cues and food choices. We began to study the early mechanisms of cognitive processing, but there are several additional steps between attentional processing of environmental cues and food choices. Measuring other effects of priming on attentional processes and cognitive biases could contribute to explain why some people adopt unhealthy diets. This work provides perspectives on how the omnipresence of subliminal food cues in our current environment could affect individuals with varying weight statuses differently. While it is difficult to generalize such findings, they open a path toward further research on how food cues from the environment can influence food choices, leading to suboptimal food choices. Improving our knowledge of the cognitive factors involved in obesity will undeniably give us a better understanding of how to prevent people from making unhealthy food choices, and, consequently, improve our ability to promote health through diet.

## Data Availability

All datasets generated for this study are included in the manuscript and/or the Supplementary Files.

## Ethics Statement

The study was conducted in accordance with the Declaration of Helsinki and was approved by the local ethical committee (Institutional Review Board de l’Inserm- CEEI- number file: IRB00003888, IORG0003254, FWA00005831). This research study adhered to all applicable institutional and governmental regulations concerning the ethical use of human volunteers.

## Author Contributions

MM designed the experiment and collected the data. She completed the statistical analysis with the help of CC (statistician) and drafted the manuscript. M-CB assisted with recruitment at Dijon public hospital. SC helped designing the experimental method and drafted the manuscript. All the authors contributed to the interpretation of the findings and critical revision of the manuscript. All the authors read and approved the final manuscript.

### Conflict of Interest Statement

The authors declare that the research was conducted in the absence of any commercial or financial relationships that could be construed as a potential conflict of interest.

## Supplementary Material

SUPPLEMENTARY FIGURE 1 Raw data for implicit condition, with zoom.

SUPPLEMENTARY FIGURE 2 Raw data for explicit condition, with zoom.
